# Value-based healthcare payment models: a wolf in sheep’s clothing for patients and clinicians

**DOI:** 10.1080/07853890.2024.2382948

**Published:** 2024-07-24

**Authors:** Forrest Bohler, Allison Garden, Callaham Brock, Lily Bohler

**Affiliations:** a Oakland University William Beaumont School of Medicine, Rochester, MI, USA; b Edward Via College of Osteopathic Medicine - Carolinas Campus, Spartanburg, SC, USA; c MT State University, Bozeman, MT, USA

**Keywords:** Value-based healthcare, health insurance, preventative medicine, population health, insurance reform, fee-for-service, physician reimbursement, patient outcome, healthcare efficiency

## Abstract

Value-based healthcare payment models are an alternative insurance payment system that compensates healthcare providers based on their patients’ outcomes rather than the individual services healthcare workers provide. This shift from the current fee-for-service model that predominates our medical system has received renewed popularity and attention within organized medicine such as the American Medical Association. Advocates believe that this new payment model will address many of the unsolved issues in healthcare such as medical waste and unsustainable healthcare costs. In practice, however, this model is plagued with a myriad of unresolved issues of its own. In this commentary, we outline these issues and suggest that the intentions of those advocating for value-based payment models are either misguided or disingenuous. We then offer solutions that preserve our current fee-for-service model while making necessary changes that will benefit both physicians and patients nationwide.

## Value-based healthcare overview

Value-based healthcare (VBHC) is an alternative healthcare payment model from the current fee-for-service (FFS) model that predominates our medical system. Rather than compensating physicians directly for the services they provide patients, VBHC seeks to pay physicians based on patient outcome in an attempt to improve population health, while simultaneously lowering total healthcare costs through improved efficiency [1]. Recently, these payment models have received renewed attention and surging popularity within organized medicine [2]. While in theory VBHC may seem to be a viable alternative to FFS, there are a myriad of issues attached to this payment model that have not been readily explored. In this commentary, we outline these issues so providers can make an informed decision when considering whether or not to support this new payment model.

## The 80/20 problem

Patient outcome is at the heart of VBHC when determining physician payout. Physicians that meet pre-determined quality metrics regarding patient outcomes will subsequently receive higher reimbursements. Proponents of VBHC claim that shifting the payment incentives of physicians to this new model will encourage providers to focus more on each patient and less on total volume of services provided. In theory, this should incentivize physicians, particularly those in primary care, to promote preventative medicine in their clinic by compensating them for these practices. In turn, these preventative practices, that are not adequately reimbursed under the FFS model, will lower our country’s sick load, subsequently saving our healthcare system billions of dollars in the management of chronic illnesses.

On paper, VBHC seems to be a promising strategy to improve population health, yet neglects to account for factors that determine patient outcome. Although significant strides in modern medicine have been made, the direct actions of clinicians alone account for only 10-20% of patient outcome [3]. The other 80-90% is determined by non-clinical factors known as social determinants of health [3]. These factors, tied closely to a patient’s socioeconomic status, are largely not addressable by physicians in clinical practice. Instead, this work lies with other professionals such as social workers and politicians who can enact change on a systemic level. This paradigm then begs the question of the utility of a payment model that reimburses physicians’ work based on factors that are 80% beyond the control of their individualized efforts. Would any profession be comfortable with a payment model that determines income through factors beyond the control of the worker?

Furthermore, the success of VBHC on cost savings has yet to be demonstrated in a reliable manner given that Medicare’s Accountable Care Organizations (ACO), widely considered to be the most prominent form of VBHC, has produced only modest healthcare savings, ranging from 0.2% to 0.3% [4].

## The unintended consequences of VBHC

There are many unintended consequences that are likely to occur with the adoption of VBHC. Proponents have yet to devise an effective method to quantify the qualitative nature of patient outcome and translate this to physician payout. This issue may prove to be a non-starter for widespread adoption of VBHC, but if an implementable system is concocted, increased administrative burdens are an inevitability for physicians. Compared to FFS models, VBHC requires additional compulsory data collection on patient outcomes to adequately measure whether or not pre-determined goals are being met [5]. This data collection will likely increase physicians’ non-patient centered workload, similar to the increase in non-clinical work seen in the shift to electronic health records [6]. Considering burdensome, non-clinical work is cited as a leading cause for record high physician burnout, VBHC will likely exacerbate the physician shortage, driving more clinicians out of medicine [7]. This is further supported by findings that demonstrate at least in some cases (e.g. physicians in small practices), those participating in ACOs have higher rates of burnout compared to those not in ACOs [8]. It is possible that this burden could be alleviated by non-physician personnel but this additional support staff will likely increase overhead costs for healthcare facilities.

Additionally, health equity among already marginalized populations such as racial minorities and those in rural communities may potentially worsen under a VBHC system. If physician income is determined by patient outcome, clinicians will naturally be incentivized to practice in populations that tend to have favorable health outcomes. These patients overwhelmingly tend to be affluent, white, suburban populations. The VBHC model may subsequently lead to an avoidance of minority populations that have the greatest need for physician services.

## The true intent of VBHC

While there are theoretical benefits of VBHC for primary care, they are not readily apparent for specialist providers whose bulk of care is centered around addressing pre-existing medical conditions. For these providers, their patient population is often beyond the help of preventative medical practices. Instead, volume of care provided to this population, in light of the looming physician-shortage, is key to improving health outcomes. A VBHC model makes little sense for these providers, as specialists will no longer be incentivized to treat a large volume of patients. However, proponents insist VBHC must be expanded to include specialist care, as the costs associated with their services constitute a significant portion of our healthcare expenditures [9]. While this may be true when considering money allocated to physician reimbursements in isolation, costs associated with physician services comprise a mere 14.9% of the country’s total healthcare expenditures [10]. Herein lies the more plausible intentions of VBHC: lower healthcare expenditure by decreasing physician reimbursement under the guise of cost-savings *via* more efficient healthcare practices. Suggesting payments related to physician services are the primary drivers of our nation’s bloated healthcare expenses is intellectually dishonest and should raise alarm for an ulterior motive for promoting VBHC.

## The solution

If we were serious as a country about reducing healthcare costs through improved efficiency and preventative medicine practices, we would start with significant reform of our current system. If we want to incentivize primary care physicians to spend more time with patients, we must increase their baseline payments for routine services so that they are adequately compensated and do not feel the need to increase their volume, a natural response to the year-over-year cuts in Medicare reimbursement. In January alone, Medicare implemented a 1.69% cut in physician reimbursement [11]. When adjusted for inflation, total physician reimbursement from Medicare has declined by 30% since 2001 [11]. During this same time, the costs of medical school tuition has risen drastically, outpacing inflation, as the median indebtedness of a newly graduated medical student is $200,000 [12,13]. In order to offset these financial issues, increasing physician reimbursement is necessary. We speculate, however, that these pay increases are likely widely unpopular among VBHC advocates and antithetical to their underlying motives.

Many physicians have also increased the practice of defensive medicine in response to anxiety and fears of malpractice litigation [14]. Defensive medicine is generally regarded as medical practices that deviate from the standard of care in order for a provider to protect themself from litigation from patients [15]. Its estimated that costs associated with defensive medical practices ranges from $650 to $850 billion annually [16]. Often times this results in high levels of unnecessary medical testing and imaging, resulting in increased waste in healthcare [17]. In an attempt to reduce this level of spending, some states, such as Montana, require that the merits of potential malpractice suits be first reviewed and approved by the Montana Medical Legal Panel before plaintiffs are allowed to file a lawsuit in court [18]. Policies such as these protect healthcare workers from frivolous lawsuits and likely lead to lower rates of defensive medicine practices. This likelihood is underscored by findings that show a 5% reduction in inpatient spending with no compromises to patient outcome for who enjoy medical liability immunity as seen in federally employed physicians [19]. Legislation at both the state and federal level to extend this level of protection to physicians nationwide would likely result in similar reductions in spending.

Additionally, if we want to lower our country’s healthcare expenditure and improve patient outcome, we must implement solutions that address the root cause of these problems: chronic illness. It’s laughable to suggest physician behavior in the clinic is the primary driver of worsened patient outcomes and unsustainable expenditures. It is no secret that the standard American diet is one of, if not the most, significant causes of our country’s disease burden. Federal dietary guidelines such as the Food Pyramid and MyPlate were overwhelming failures in promoting healthy eating patterns. It’s befuddling to consider America is one of the only countries in the world that allows highly processed foods, such as soda, to be purchased with food assistance programs [20]. Why then are we subsiding the very cause of the problem we are attempting to solve and blaming the FFS model for the current state of our medical expenditures? We wouldn’t allow recipients of government assistance to purchase a pack of cigarettes with taxpayer dollars and then point the finger at pulmonologists for increasing costs associated with lung cancer, telling them we need radical reform to change how we pay them. How is this any different?

## Data Availability

This commentary did not generate any new data and only utilized existing publications that can be found in the References section below.
